# Renal erythropoietin-producing cells in health and disease

**DOI:** 10.3389/fphys.2015.00167

**Published:** 2015-06-03

**Authors:** Tomokazu Souma, Norio Suzuki, Masayuki Yamamoto

**Affiliations:** ^1^Department of Medical Biochemistry, Tohoku University Graduate School of MedicineSendai, Japan; ^2^Division of Interdisciplinary Medical Science, United Centers for Advanced Research and Translational Medicine, Tohoku University Graduate School of MedicineSendai, Japan; ^3^Division of Nephrology and Hypertension, Feinberg School of Medicine, Northwestern UniversityChicago, IL, USA

**Keywords:** erythropoietin, fibrosis, hypoxia, plasticity, renal Epo-producing cell (REP)

## Abstract

Erythropoietin (Epo) is an indispensable erythropoietic hormone primarily produced from renal Epo-producing cells (REPs). Epo production in REPs is tightly regulated in a hypoxia-inducible manner to maintain tissue oxygen homeostasis. Insufficient Epo production by REPs causes renal anemia and anemia associated with chronic disorders. Recent studies have broadened our understanding of REPs from prototypic hypoxia-responsive cells to dynamic fibrogenic cells. In chronic kidney disease, REPs are the major source of scar-forming myofibroblasts and actively produce fibrogenic molecules, including inflammatory cytokines. Notably, myofibroblast-transformed REPs (MF-REPs) recover their original physiological properties after resolution of the disease insults, suggesting that renal anemia and fibrosis could be reversible to some extent. Therefore, understanding the plasticity of REPs will lead to the development of novel targeted therapeutics for both renal fibrosis and anemia. This review summarizes the regulatory mechanisms how hypoxia-inducible *Epo* gene expression is attained in health and disease conditions.

## Introduction

Erythropoietin (Epo) is an indispensable erythropoietic glycoprotein hormone that induces red blood cell production (Haase, 2010; Bunn, 2013; Suzuki, 2015). Circulating Epo concentration is dynamically altered by the presence of hypoxia or anemia; up to 1000-fold increase in the circulating Epo concentration is reported in anemic patients (Bunn, 2013). Epo exerts its erythropoietic function through binding to Epo receptor (EpoR) (Remy et al., 1999). The EpoR expression level in erythroid progenitor cells is dependent on their differentiation stage; EpoR is most highly expressed in late-stage erythroid progenitors to early erythroblasts (Suzuki et al., 2003; Yamazaki et al., 2013). The Epo–EpoR signaling primarily mediates survival signaling in these progenitor cells and prevents their apoptosis, causing proliferation and differentiation, and thereby activating erythropoiesis (Wu et al., 1995).

Regarding its application to medicine, Epo represents a prototypical success of molecular biology. The presence of Epo was first suggested in the nineteenth century based on the high blood viscosity of people living in or returning from high altitude areas (Koury, 2005; Bunn, 2013). Experimentally, the presence of Epo as an erythropoietic humoral factor was discovered in the early twentieth century due to the erythropoietic property of serum from phlebotomized rabbits. Most notably, in 1977, Miyake et al. purified Epo from 2550 liters of urine from patients of aplastic anemia and determined its amino acid sequence (Miyake et al., 1977). Based on this finding, the human *Epo* genes were cloned in 1985 (Jacobs et al., 1985; Lin et al., 1985). Then, recombinant human Epo (rHuEpo) was successfully used to treat anemic patients with end-stage renal diseases (ESRD) (Bunn, 2013). Furthermore, studies on *Epo* gene regulation led to the identification of hypoxia-inducible transcription factors (HIFs) and hypoxia response elements (HREs) as the HIF-binding consensus sequence on genome (Semenza et al., 1991b), and to the current understanding of the molecular mechanisms of cellular adaptation to hypoxia (Semenza, 2011; Ratcliffe, 2013).

In past decades, many researchers have made rigorous efforts to identify erythropoietin-producing cells in kidneys; however, a uniform understanding of which cells produce Epo in kidneys was not established until the era of genetically modified mice (Suzuki et al., 2007). Using gene targeting and bacterial artificial chromosome (BAC) transgenic methods, we identified nearly all of interstitial fibroblast-like cells in the cortex and outer medulla to be renal erythropoietin-producing cells (REPs) (Obara et al., 2008; Pan et al., 2011; Yamazaki et al., 2013). Furthermore, interests in REPs have markedly increased by the evidence showing the crucial link between fibrosis and anemia *via* the loss of Epo-producing ability of myofibroblast-transformed REPs (MF-REPs) (Maxwell et al., 1997; Asada et al., 2011; Souma et al., 2013). Importantly, this direct link indicates that renal fibrosis and anemia could be simultaneously treated by targeting or regulating the cellular properties of REPs. In this review, we provide a summary of recent lines of evidence regarding the role of REPs in health and disease and discuss future research directions for regulating REP functions to treat both fibrosis and anemia.

## Identification of renal erythropoietin-producing cells

To identify Epo-producing cells in the kidneys, *in situ* hybridization and/or immunohistochemistry have been utilized to detect Epo in tissue sections. However, these strategies have limited sensitivity and specificity for detecting Epo, hampering the establishment of a consensus as to which cells produce Epo. By using a transgenic mouse technology, SV40 T antigen cDNA was integrated into the *Epo* gene locus to identify renal Epo-producing cells (REPs) with anti-T antigen antibodies (Maxwell et al., 1993), and the results showed that renal fibroblasts are the top candidates among the proposed Epo-producing cells including tubular epithelial cells, glomerular mesangial cells, and interstitial fibroblasts.

To unequivocally determine the identity of REPs, we have utilized two complimentary strategies, BAC transgenic mice and green fluorescent protein (GFP) reporter knock-in mice (Obara et al., 2008; Pan et al., 2011). The BAC transgenic mouse lines (Tg-EpoGFP) harbor transgene constructs, which direct GFP expression under the control of the 180-kb regulatory region around the mouse *Epo* gene, and label Epo-producing cells by GFP expression with high sensitivity. The other strategy using a genetically modified mouse line (KI-EpoGFP, *Epo*^*GFP/wt*^), in which the GFP cDNA is knocked-in to the endogenous *Epo* gene, assures a higher specificity than the Tg-EpoGFP strategy because the GFP expression in the knock-in mice is directed by endogenous *Epo* gene regulation. Only a few renal interstitial cells are labeled by both strategies under normal conditions, whereas GFP-positive cells robustly emerge and increase in the kidneys under anemic or hypoxic stress. These GFP-positive cells in the interstitium are fibroblast-like cells expressing neural genes [e.g., microtubule-associated protein 2 (Map2), nerve growth factor receptor (Ngfr), and neurofilament light peptide (Nefl)]. We named these cells renal erythropoietin-producing cells, or REPs (Suzuki et al., 2007; Obara et al., 2008).

## Epo gene modification for further analyses of REPs

*Epo*-knockout mice die at approximately embryonic day 12.5 (E12.5) due to severe anemia (Wu et al., 1995), hampering the analyses of *Epo* gene function in adults. We assumed that the embryonic lethality of *Epo*-null mice could be rescued by transgenic *Epo* gene expression in the livers of embryos because hepatocytes are the Epo-producing cells at the lethal time point (Suzuki et al., 2011). To this end, we have utilized the knowledge regarding *Epo* gene regulation; i.e., the proximal downstream region of the *Epo* gene transcription end site (*EpoHE*, hepatic enhancer) is sufficient for *Epo* gene expression from hepatocytes, but is dispensable for renal *Epo* gene expression (Suzuki et al., 2011). An 8-kb *Epo* transgene (*Tg-Epo*^3.3^) containing *EpoHE* but not the kidney regulatory elements has been constructed. This transgene successfully rescues the embryonic lethality of *Epo*-null mice (*Epo*^*GFP/GFP*^) (Yamazaki et al., 2013). In the transgene-rescued mice (named as Inherited Super-Anemic Mice, ISAM; *Epo*^*GFP/GFP*^*:Tg-Epo*^3.3^), Epo is mainly produced in their liver, and the plasma Epo concentrations are repressed to undetectable levels by the weaning age, which is the ontogenetic time point at which the Epo-producing site switches from the liver to the kidney. ISAM develop adult onset severe chronic anemia due to defect in renal Epo production, and Epo-producing cells are efficiently labeled with GFP expression from the *Epo* gene locus in adult ISAM kidneys (Yamazaki et al., 2013).

**Figure 1:**
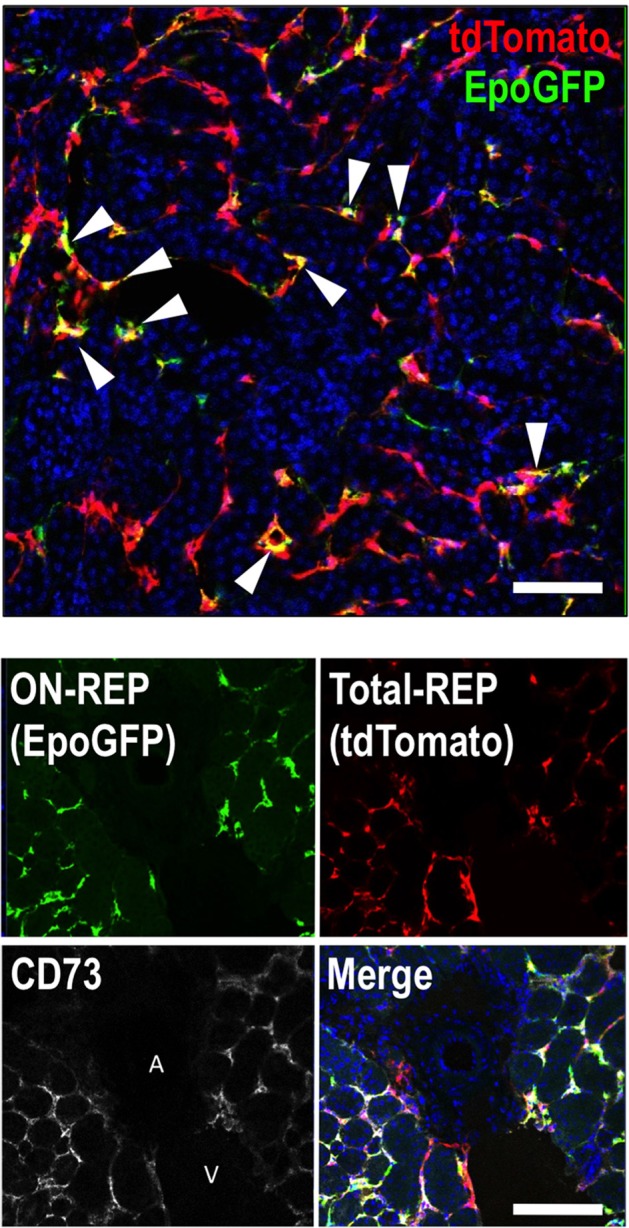
**REPs are peri-capillary CD73-positive fibroblast-like cells**. A kidney section from ISAM-REC (ISAM:R26RtdTomato:Tg-EpoCre) shows ON-REPs expressing EpoGFP (arrowheads, positive for both green and red) in the total REP population (positive for tdTomato fluorescence, red). The nuclei are stained by DAPI (blue). A kidney section from ISAM-REC shows that REPs or CD73-positive fibroblasts are distributed in peri-capillary interstitial spaces but not in peri-arterial interstitial areas of the kidneys. DAPI (blue) is used for nuclear staining in the merged image. Abbreviations: A, artery; V, vein. Scale bar, 100 μm.

The numbers of GFP-expressing REPs are markedly higher in ISAM with chronic severe anemia than those of Tg-EpoGFP or KI-EpoGFP mice with bleeding-induced acute anemia (Yamazaki et al., 2013). Thus, it appears that the EpoGFP-expressing cells represent a small portion of the total REPs. To identify the total REPs, we have conducted fate-tracking assays of cells with a history of Epo production using newly generated Tg-EpoCre mice in which the Cre-recombinase is expressed under the regulation of the 180-kb transgene containing the *Epo* gene regulatory region (Souma et al., 2013; Yamazaki et al., 2013). To detect EpoCre-labeled cell lineages, we have utilized the R26RtdTomato mouse line in which cells that have expressed Cre at least once are marked by tdTomato red fluorescence. When *EpoCre* transgene expression is enhanced by chronic severe anemia in ISAM (ISAM:R26RtdTomato:Tg-EpoCre, ISAM-REC), tdTomato fluorescence is detected in almost all fibroblast-like interstitial cells (PDGFRβ^+^CD73^+^ cells) in the cortex and outer medulla (both the outer stripe and the inner stripe) (Figure 1). The results indicate that cells capable of producing Epo (total REPs) are far more abundant in kidneys than we previously expected in Tg-EpoGFP mice. Interestingly, we have observed REPs around the peritubular capillaries, but not in the regions surrounding the larger vessels (Figure 1). Furthermore, in ISAM-REC kidneys, approximately 10% of tdTomato-positive cells express EpoGFP (ON-REPs), indicating that most REPs (OFF-REPs) are resting from Epo production even under severe anemic conditions (Figure 1) (Yamazaki et al., 2013; Souma et al., 2013).

## Regulatory mechanisms governing Epo synthesis

The balance between oxygen supply and demand precisely controls renal Epo production. A salient physiological study using isolated rat kidneys with hypoxic perfusate shows that renal Epo secretion is regulated by tissue oxygen tension (Pagel et al., 1990). Distant organs, such as skin have been found to participate in regulation of renal Epo synthesis through sensing a hypoxic atmosphere (Boutin et al., 2008). Recently, Dimke et al. demonstrated that the importance of the renal oxygen levels for renal *Epo* gene expression using a mouse model with tubular-specific *Vegfa* (vascular endothelial growth factor a) gene deletion. In this mouse model, the kidney has scarce vascularization, resulting in renal hypoxia, increased Epo secretion, and severe polycythemia (Dimke et al., 2015), indicating that the oxygen supply in the kidneys, but not in other organs or tissues, is the most important factor to determine renal Epo synthesis.

**Figure 2:**
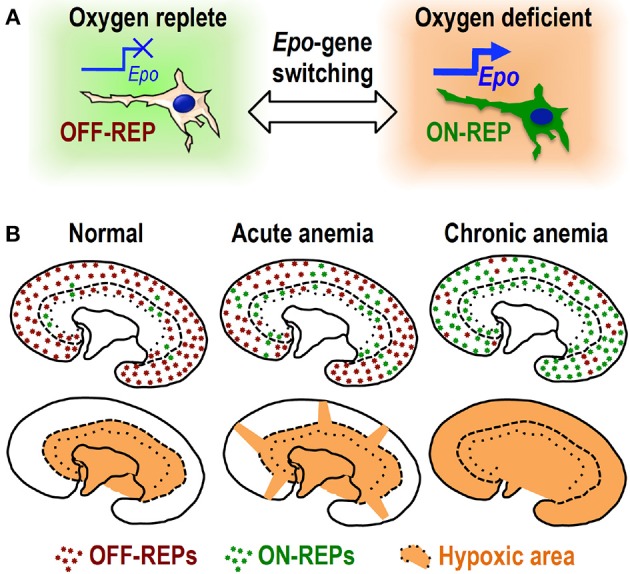
**ON–OFF regulation of Epo synthesis in REPs. (A)** Oxygen deficiency turns OFF-REPs into ON-REPs by turning on “*Epo* gene switch.” **(B)** REPs are primarily in the “OFF” state (OFF-REPs, brown dots) under normal oxygen conditions. Under acute anemic conditions, some clusters of OFF-REPs turn into ON-REPs (green dots). The distribution of ON-REPs extends toward hypoxic cortical areas (orange). Strikingly, most OFF-REPs turn into ON-REPs under chronic anemia. This recruitment of ON-REPs determines the total amount of Epo secreted by the kidneys.

Induction of hypoxia/anemia widely spread the distribution of currently Epo-producing cells (ON-REPs: defined by EpoGFP expression) from the juxta-medullary region, which is physiologically hypoxic, to the entire cortex (Koury et al., 1989; Eckardt et al., 1993; Obara et al., 2008; Souma et al., 2013; Yamazaki et al., 2013). Interestingly, the ratio of ON-REPs to the total REPs in the kidney correlates well with plasma Epo levels, indicating that renal Epo secretion is regulated by “ON–OFF switch” for *Epo*-gene expression in each REP in a hypoxia-inducible manner (Figure 2). In other words, the conversion of OFF-REP to ON-REP is governed by transcriptional control of the *Epo* gene, which is primarily regulated by HIF transcription factors (Haase, 2010; Suzuki, 2015).

**Figure 3:**
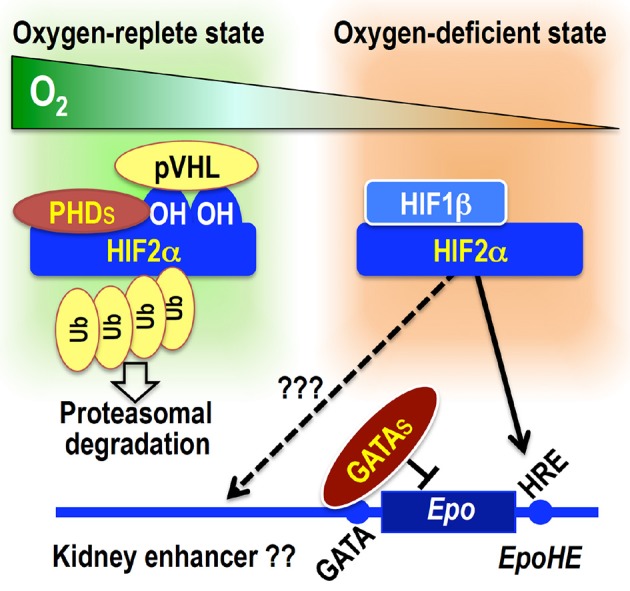
**Overview of** ***Epo*** **gene transcriptional regulation**. Under oxygen-replete conditions, PHDs hydroxylate HIF2α proteins, leading to pVHL-mediated ubiquitination and degradation of HIF2α. Under oxygen-depleted condition, PHDs are inactivated, and HIF2α escapes degradation. The HIF2α–HIF1β heterodimer activates *Epo* gene transcription by binding to the HRE. The functional HRE for hepatic *Epo* gene expression (*EpoHE*) is in the proximal downstream region of the transcription end site, whereas a kidney enhancer has been suggested to be located in a region far upstream of the transcription start site. GATA factors constitutively suppress ectopic *Epo* gene expression in epithelial lineage cells by binding to the promoter GATA sequence.

Understanding of the *Epo* gene regulation has been advanced by the discovery of Epo-producing hepatoma cell lines (Hep3B and HepG2) (Goldberg et al., 1987). Analyses of these cells led to the discovery of HIF proteins and their binding sequences, HREs (Figure 3) (Semenza et al., 1991b). In order to understand renal *Epo* gene regulation, transgenic mouse lines with different regulatory regions have been generated. For instance, *EpoHE* containing an HRE is proved to be necessary and sufficient to direct hepatic *Epo* gene expression *in vivo* (Suzuki et al., 2011). However, this enhancer is dispensable for renal *Epo* gene expression, indicating that other *cis*-elements direct renal Epo production (Figure 3). *EpoHE* also contains a direct repeat sequence that is considered a binding site for hepatic nuclear factor 4 (HNF4) and/or retinoid X receptor (RXR); together with HIFs, these factors synergistically regulate liver-specific, hypoxia-inducible *Epo* gene expression (Galson et al., 1995; Makita et al., 2001).

One of the important lingering questions about *Epo* gene regulation is the location and characteristics of the regulatory regions for renal *Epo* gene expression. It has been surmised that an essential regulatory region may lie far upstream from the transcription start site (−14 to −6 kb; Figure 3) (Semenza et al., 1991a; Madan et al., 1995; Suzuki et al., 2013). We also have utilized a series of BAC transgenic reporter lines that direct GFP expression from the transgenic *Epo* locus, and the regulatory region for renal *Epo* gene expression has been narrowed down to the region from −17 to +15 kb (Obara et al., 2008; Suzuki et al., 2013).

The *Epo* gene promoter lacks the typical TATA box, but has a negative regulatory element with a GATA box (Imagawa et al., 1991). By mutating the GATA box in *EpoGFP* transgene constructs, the GATA box is proved to be not necessary for inducible *Epo* gene expression in either REPs or hepatocytes (Obara et al., 2008). However, the GATA box is turned out to be indispensable for repressing ectopic *Epo* gene expression in epithelial lineage cells, including distal tubular cells, bronchial epithelial cells, and cholangiocytes. In renal distal tubular cells, the GATA box is occupied by GATA transcription factors. In this manner, the GATA box contributes to the tissue-specific *Epo* gene expression (Figure 3) (Obara et al., 2008).

Of the transcription factors interacting with these *Epo* gene regulatory regions, HIFs play the central role in hypoxia-inducible *Epo* gene expression (Haase, 2010; Suzuki, 2015). HIFs are heterodimeric complexes comprising one α subunit (HIF1α, HIF2α, and HIF3α) and one β subunit (HIF1β, also known as ARNT) (Semenza, 2011; Ratcliffe, 2013). Under oxygen-replete conditions, the proline residues of HIFα subunits are hydroxylated by HIF prolyl hydroxylases (or prolyl hydroxylase domain proteins, PHDs: PHD1, PHD2, and PHD3), leading to their proteasomal degradation *via* pVHL (von Hippel Lindau protein)-mediated ubiquitination (Figure 3) (Tanimoto et al., 2000). Under oxygen-deficient conditions, the enzymatic activities of PHDs are inhibited, and HIFα proteins escape from degradation. Stable HIFα proteins dimerize with HIF1β, and the heterodimeric complexes bind to HRE in regulatory regions of target genes, including *Epo* (Figure 3). PHD2 and HIF2α are the most important factors among those related to PHD-HIF signaling in the renal Epo production (Table 1) (Souma et al., in press).

**Table 1:** **Summary of gene targeting studies of hypoxia-related factors on renal** ***Epo*** **gene regulation**.

**Deleted factor**	**Effects on renal *Epo* gene expression**	**References**
HIF1α	Inducible KO in adult: no change	Gruber et al., 2007
HIF2α	Inducible KO in adult: decrease Kidney-specific KO: decrease	Gruber et al., 2007; Rankin et al., 2006
PHD1 and PHD3	Systemic double KO: no change	Takeda et al., 2008
PHD2	Inducible KO in adult: increase REP-specific KO: increase	Takeda et al., 2008; Souma et al., in press
pVHL	Kidney-specific KO: increase	Rankin et al., 2006

*KO, knockout*.

In addition to PHDs, another HIF-hydroxylase, FIH-1 (factor inhibiting HIF-1), is involved in the cellular responses to hypoxia (Mahon et al., 2001). FIH-1 negatively regulates HIF-dependent transactivation by inhibiting CREB-binding protein (CBP)/p300 recruitment to HIFα *via* the asparaginyl hydroxylation of HIFα proteins. The asparaginyl hydroxylation is more resistant to suppression by hypoxia than the prolyl hydroxylation (Tian et al., 2011). FIH-1-dependent hydroxylation is more prone to be inhibited by oxidative stresses than by hypoxic stresses (Masson et al., 2012). Consistent with these findings, the systemic knockout of FIH-1 does not result in defects in the *Epo* gene regulation directly (Table 1) (Zhang et al., 2010).

## REPs as major contributors to renal fibrosis

All chronic nephropathies progress with tubular atrophy and interstitial fibrosis along with the relative loss of Epo production (Quaggin and Kapus, 2011). While fibrosis is an essential biological process for repairing tissue injuries, uncontrolled and persistent injuries lead to sustained fibrogenesis, followed by destruction of tissue architecture and organ failure (Quaggin and Kapus, 2011; Friedman et al., 2013). Therefore, the identification of therapeutics controlling the pathological fibrogenic response would be beneficial for many devastating diseases, such as chronic kidney disease (CKD), cirrhosis, and pulmonary fibrosis (Friedman et al., 2013). Since a strong correlation between tubulo-interstitial injury and decreased glomerular filtration rate was first described, many researchers have sought the origin of scar-forming cells, i.e., myofibroblasts that are fibroblast-like cells with contractile properties, in the renal interstitium (Quaggin and Kapus, 2011; Boor and Floege, 2012). Using genetic lineage tracing, various cellular sources have been postulated as the origins of myofibroblasts, including pericytes, resident fibroblasts, tubular cells, endothelial cells, fibrocytes, and bone marrow-derived cells, but exact contributions of the sources to renal fibrosis still remain under debate (summarized in Table 2) (Quaggin and Kapus, 2011; Mack and Yanagita, 2014).

**Table 2:** **Summary of the origins of renal myofibroblasts**.

**Cell fate tracking method**	**Target cell type**	**Injury model**	**αSMA^+^ cells**	**References**
**TUBULAR EPITHELIAL CELLS (EPITHELIAL–MESENCHYMAL TRANSITION, EMT)**				
γGT-Cre	Cortical tubular cells	UUO	36%	Iwano et al., 2002
γGT-Cre	Cortical tubular cells	UUO	5%	LeBleu et al., 2013
Pax8-rtTA:TetON-Cre	All tubular cells	TGFβ O/E	0%	Koesters et al., 2010
Ksp-Cre	Distal tubular cells	UUO	0%	Li et al., 2010
Six2-Cre/Hoxb7-Cre	All tubular cells	UUO	0%	Humphreys et al., 2010
**INTERSTITIAL MESENCHYMAL CELLS (FIBROBLASTS/PERICYTES/REPS)**				
FoxD1-Cre	Pericytes, fibroblasts	UUO	>90%	Humphreys et al., 2010
P0-Cre	Fibroblasts	UUO	93%	Asada et al., 2011
EpoCre	REPs	UUO	>80%	Souma et al., 2013
**ENDOTHELIAL CELLS (ENDOTHELIAL-MESENCHYMAL TRANSITION, ENDMT)**				
Tie2-Cre	Endothelial cell	UUO/STZ/Alport	30–50%	Zeisberg et al., 2008
Cdh5-Cre	Endothelial cell	UUO	10%	LeBleu et al., 2013
**BONE MARROW-DERIVED CELLS**				
Fsp1-EGFP, BMT	Myeloid cells, fibroblasts	UUO	15%^**^	Iwano et al., 2002
Col1a1-GFP, BMT	Collagen-producing cells	UUO	<0.1%	Lin et al., 2008
Y chromosome, BMT	Bone marrow-derived cells	UUO	8.6%^*^	Roufosse et al., 2006
Y chromosome, BMT	Bone marrow-derived cells	UIRI	Detected	Lin et al., 2009
R26-hPAP, BMT	Bone marrow-derived cells	UIRI	32%^**^	Broekema et al., 2007
αSMA-RFP, BMT	αSMA-positive cells	UUO	35%	LeBleu et al., 2013

*UUO, unilateral ureteral obstruction; UIRI, unilateral ischemia reperfusion injury; STZ, streptozotocin-induced diabetic nephropathy; Alport, Col4a3 knockout mice; O/E, overexpression*.

* No collagen I production;

** collagen I production; ^**^12% of αSMA-positive cells in normal kidneys were derived from the bone marrow.

A possible direct link between the loss of Epo production and progression of fibrosis was first proposed in 1997 (Maxwell et al., 1997). Maxwell et al. showed that REPs, which are tagged by integrated SV40 T antigen cDNA in the *Epo* gene locus, turn into desmin-positive myofibroblasts following ureteral obstruction injury, and that the number of T-antigen-expressing cells decreases to less than 5% of control kidneys in 9 days after the obstruction (Maxwell et al., 1997). Interestingly, tamoxifen, a selective estrogen receptor modulator, is found to improve the Epo-producing ability of myofibroblasts (Asada et al., 2011).

**Figure 4:**
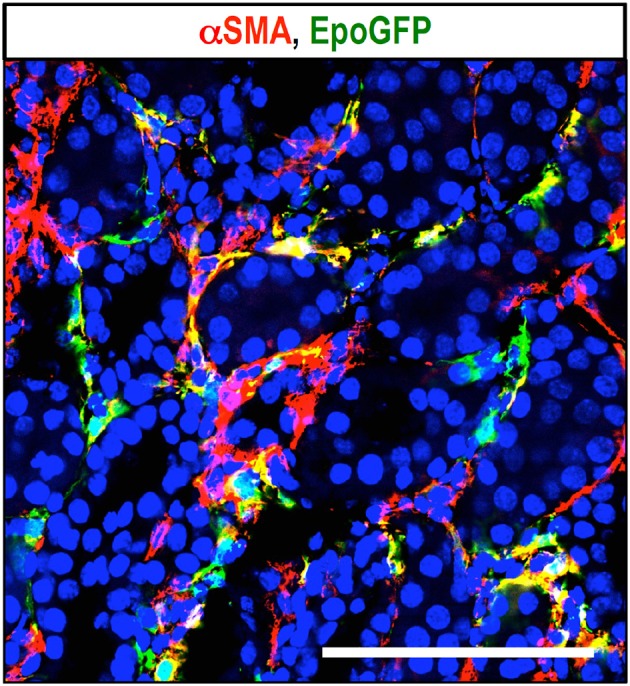
**Myofibroblast transformation of REPs**. Immunohistochemical detection of myofibroblasts with αSMA antibody (red) in ureteral obstructed kidney of ISAM. EpoGFP-positive REPs (green) transform into myofibroblasts (MF-REPs) upon kidney injury induced by ureteral obstruction for 3 days. Blue: DAPI for nuclear staining. EpoGFP protein expression does not reflect ongoing *Epo* gene transcription due to its longer half-life. *EpoGFP* mRNA expression is immediately silenced by urinary obstruction (Souma et al., 2013). Scale bar, 100 μm.

To better understand the contribution of REPs to renal fibrosis and the link between fibrosis and anemia, ISAM have been utilized as the most efficient reporter mouse model for the Epo-producing ability. The Epo-producing ability of REPs is lost in kidneys within 24 h after ureteral obstruction, and strong α-smooth muscle actin (αSMA) expression is observed in REPs from 2 days following ureteral obstruction onward (Figure 4). These results indicate that the renal fibrogenic milieu strongly represses *Epo* gene transcription in REPs during their myofibroblast transformation process (Souma et al., 2013). Gene expression analyses of isolated MF-REPs show that MF-REPs produce inflammatory cytokines, chemokines, and extracellular matrix; all of which drive renal fibrosis. Consistent with the finding that renal myofibroblasts contribute to the inflammatory milieu, damage-associated molecular patterns (DAMPs) induce IL-6 and MCP1 productions in myofibroblasts (Campanholle et al., 2013). Additionally, the loss of local Epo production might have deteriorating effects on fibrogenesis and inflammation in the kidneys, because cytoprotective function of Epo beyond erythropoiesis has been predicted (Noguchi et al., 2008).

Functional lineage tracing using the *EpoCre* transgene shows that the cortical and outer medullary interstitium of ISAM-REC kidneys are primarily replaced by MF-REPs in unilateral ureteral obstruction (UUO) model, indicating that REPs are the major source of the myofibroblasts (Table 2) (Souma et al., 2013). MF-REPs lose their Epo-producing ability and persist in scar tissues. These results indicate that the transformation of REPs to MF-REPs or myofibroblasts directly links both fibrosis and anemia. Interestingly, the product of the hemoglobin (Hb) concentration times the Epo concentration in the peripheral blood of patients with diabetic nephropathy correlates well with the stages of diabetic nephropathy and predicts future chronic renal failure in overt diabetic nephropathy (Inomata et al., 1997). Although confirmation of this argument waits for larger studies, we surmise that Epo would be a good biomarker to estimate the severity of interstitial injury and to predict the prognosis of damaged kidneys based on the short half-life (4–8 h) of Epo (Jelkmann, 2002).

One aspect that makes the determination of the origins of myofibroblasts difficult is the complexity regarding the identity of the interstitial cells, i.e., pericytes, fibroblasts, and REPs. It has been shown that FoxD1-tagged pericytes are the major source of renal myofibroblasts (Humphreys et al., 2010), whereas another groups argue that resident fibroblasts are the major source of renal myofibroblasts (Table 2) (Asada et al., 2011; LeBleu et al., 2013). As mentioned above, we found that REPs are the major source of renal myofibroblasts through EpoCre-based functional lineage tracing (Souma et al., 2013). Because these three cell types (pericytes, resident fibroblasts, and REPs) share similar cellular surface markers (PDGFRβ and CD73), locations, and morphology, we believe that these cells are largely overlapping populations. Recently, Kramann et al. reported that a small subset of pericytes (Gli1^+^PDGFRβ^+^CD73^−^ cells; 0.2% of renal PDGFRβ^+^ cells), which displays mesenchymal stem cell features, is the major contributor of renal fibrosis through rigorous proliferation upon injury (Kramann et al., 2015). These evidence raise a possibility that renal myofibroblasts are originated from a Gli1^+^ subset of EpoCre-tagged REPs, and this hypothesis waits for future confirmation.

## Plasticity of REPs

**Figure 5:**
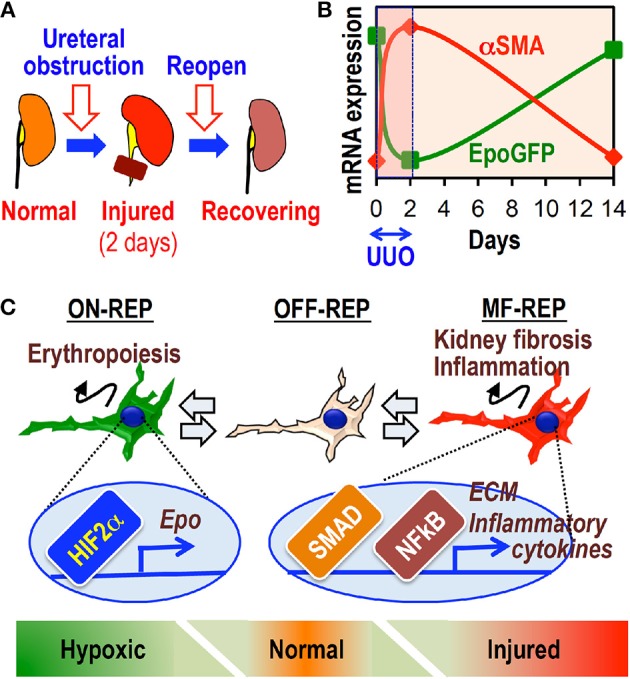
**The cellular plasticity of REPs governs both fibrosis and anemia. (A)** Schematic diagram of the reversible UUO model. The left ureter is obstructed by a vascular clip for 2 days and then re-opened afterwards. **(B)** The inverse relationship between EpoGFP and αSMA mRNA expression in whole kidneys of ISAM during reversible UUO treatment. **(C)** Schematic summary showing the plasticity of REPs. MF-REPs produce extracellular matrix (ECM) and inflammatory cytokines in injured kidneys through SMAD and NFκB signaling cascades. After the resolution of environmental cues, MF-REPs revert to their original and physiological Epo-producing phenotype. Switching between ON-REPs and OFF-REPs is determined in a hypoxia-dependent manner *via* HIF2α activation.

Some clinical and experimental reports have shown that renal structural damage, including fibrosis, is reversible (Zeisberg et al., 2003; Fioretto et al., 2006). Clinical observations have shown that more than 10% of patients on dialysis become anemia free and rHuEpo independent (Takeda et al., 2002; Kuo et al., 2005; Schwartz et al., 2005). These lines of evidence suggest that MF-REPs retain functional reversibility even in ESRD. Indeed, a short-term reversible UUO model demonstrates that MF-REPs regain their physiological characteristics, morphology, and Epo-producing ability following disease resolution (Figure 5) (Souma et al., 2013). Furthermore, dexamethasone facilitates this reversion, possibly through enhancing the resolution of inflammation in injured kidneys (Souma et al., 2013). These results indicate that REPs possess plasticity in response to environmental cues. Consistent with this observation, hepatic myofibroblasts can revert to their normal cellular character (hepatic stellate cells) during the regression of fibrosis (Kisseleva et al., 2012).

## Environmental cues for myofibroblast transformation

A genome-wide transcriptome analysis of sham-treated kidneys, obstructed kidneys, and recovering kidneys indicates that the atherosclerotic and acute phase response signals are the top two up-regulated pathways and that valine, leucine, and isoleucine degradation and fatty acid metabolisms are the top two down-regulated pathways (Souma et al., 2013). Recent transcriptome analyses using kidney samples from human CKD patients reveals that the gene expressions of fatty acid metabolism are decreased and inflammatory signaling is increased in kidney diseases (Kang et al., 2015). Based on the fact that metabolic intermediates play an important role in gene regulation, it is of great interest to determine whether deranged fatty acid metabolism would affect Epo production and whether correcting the metabolism, e.g., by PPARα activation (Kang et al., 2015), would restore Epo-producing ability of MF-REPs.

Uremic toxins are a group of compounds that are normally excreted by healthy kidneys, but accumulate upon kidney injuries. Of the toxins, indoxyl sulfates cause renal inflammation and repress Epo production by decreasing HIF-α accumulation in response to hypoxic stimuli (Chiang et al., 2011), and the NO antagonist *N*^G^-monomethyl-L-arginine (L-NMMA) represses *Epo* gene transcription through GATA2 upregulation (Tarumoto et al., 2000). These results further emphasize the importance of correcting the unbalanced microenvironment in injured kidneys.

The TGFβ and NFκB signaling pathways are two major signaling pathways involved in kidney fibrosis. Of note, TGFβ but not LPS injection leads to up-regulation of genes for extracellular matrix, while LPS but not TGFβ injection represses *Epo* gene expression. These results support the notion that TGFβ signaling is the master regulator of the fibrogenic response, while the inflammatory signals are the primary regulators of Epo repression (Souma et al., 2013).

Recently, cellular communication upon kidney injuries is gaining attention, particularly communication between renal tubular cells and fibroblasts/pericytes (Grgic et al., 2012; Humphreys et al., 2013), pericytes and endothelial cells (Schrimpf et al., 2012), and infiltrating leukocytes and resident renal cells such as distal tubular cells (Fujiu et al., 2011). However, fewer efforts have been made to decipher the nexus of environmental cues by quantifying the contribution of each cell type to the fibrogenic cues. Gene expression analyses of leukocytes isolated from injured kidneys demonstrate that expressions of TNFα, TGFβ, and MMP9 are enriched in leukocytes, whereas IL-6 and MMP3 expressions are not, suggesting that the infiltrating leukocytes and other resident renal cells collaboratively create deleterious inflammatory microenvironments (Souma et al., 2013).

Epigenetic alterations, including DNA methylation, play an important role in cellular transformation. TGFβ-mediated activation of DNA methyl transferase 1 (DNMT1) has been shown to fundamentally regulate the perpetuation of fibrosis (Bechtel et al., 2010), and DNMT1 (primarily catalyzes maintenance methylation) and DNMT3b (primarily catalyzes *de nov*o methylation) expression is increased in MF-REPs (Souma et al., 2013). Interestingly, the *Epo* gene locus is highly methylated in cell lines lacking Epo-producing abilities, indicating the importance of DNA methylation for the epigenetic silencing of *Epo* gene expression (Yin and Blanchard, 2000). Thus, one would be easy to surmise that the sustained activation of inflammatory and fibrogenic signaling may alter the epigenetic code of REPs and limit the potential of reversing MF-REPs to their original state.

## Effect of hypoxia on Epo production and kidney diseases

Kidney injuries and subsequent fibrosis disrupt oxygen delivery through vascular rarefactions and excessive extracellular matrix accumulation. Increased oxygen demands also cause kidney cells to be vulnerable to hypoxia. Renal anemia further compromises the delivery of oxygen, and the anemic hypoxia has been suggested to promote kidney diseases (Nangaku, 2006). Because HIFs are the major regulators of hypoxic adaptation, augmenting HIF signaling has been attempted to treat kidney diseases (Miyata et al., 2013). A clinical trial has been reported that the pharmacological activation of HIF signaling successfully augmented Epo production in ESRD (Bernhardt et al., 2010), underscoring the feasibility of this therapeutic strategy. Actually, REP-specific knockout of PHD2 in mice induces Epo production both in healthy and fibrotic kidneys through HIF2α activation (Souma et al., in press).

Genetic activation of HIF signaling in podocytes and/or proximal tubular cells results in the worsening of kidney diseases (Ding et al., 2006; Higgins et al., 2007), whereas deletions of HIF signaling in myeloid cells, endothelial cells, and/or whole body (Kobayashi et al., 2012; Kapitsinou et al., 2014) result in improvement of renal inflammation and fibrosis. Adequacy of hypoxia signaling upon kidney injuries has been questioned recently. Immunohistochemical analyses of kidneys in a rodent AKI model have revealed that HIF1α expression is impaired in proximal tubular cells (Fahling et al., 2013). Similarly, EpoGFP expression in ISAM is repressed by ureteral obstruction despite the presence of severe anemia (Souma et al., 2013). Furthermore, the successful augmentation of Epo production in diseased kidneys by PHD inhibitors implies that the primary cause of Epo insufficiency is the inappropriately high PHD activity in diseased kidneys, despite a severely hypoxic milieu (Bernhardt et al., 2010). Collectively, we posit that the response to hypoxia is impaired or insufficient upon kidney injury through inappropriately high PHD activity (Souma et al., in press).

Epo production is inappropriately repressed despite the presence of severe hypoxia in injured kidneys. Interestingly, TNFα-treated rodents show decreased Epo-producing ability under hypoxic or anemic conditions (Nakano et al., 2004). Consistent with this observation, inflammatory cytokines elicited in damaged kidneys repress the Epo-producing ability, emphasizing the important effect of sterile inflammation on repressing the Epo-producing ability of REPs (Souma et al., 2013). Considering these data, we posit that inflammatory signals redistribute cellular oxygen levels and activate PHDs in severely hypoxic kidneys, resulting in HIF2α degradation and impairment of the *Epo* gene expression (Souma et al., in press).

## Perspectives

We propose that identifying signals that restore physiological characteristics of REPs in fibrotic kidneys will open a new avenue for treating CKD. To accomplish this goal, several barriers must be overcome. One of the highest barriers is the difficulty in handling REPs. To screen candidate pathway to treat CKD, a good readout that reflects both diseased and recovered states of kidneys is necessary. For tubular cells, KIM-1 and NGAL (Paragas et al., 2011; Humphreys et al., 2013) are the representative readouts for the injured state and are available in clinics. For myofibroblast activation, αSMA and collagen expression is currently used for monitoring fibrogenic activity. However, these markers are not perfect for testing whether cells regain their original character upon treatment. The Epo-producing ability is the important physiological feature of renal fibroblast-like cells, and its loss is the hallmark of renal myofibroblasts. Thus, developing a methodology to culture REPs *ex vivo* and to monitor their Epo-producing ability by reporter genes would provide an opportunity to perform high-throughput screening to identify novel target signals to restore MF-REPs to normal REPs.

### Conflict of interest statement

The authors declare that the research was conducted in the absence of any commercial or financial relationships that could be construed as a potential conflict of interest.

## References

[B1] Asada N. Takase M. Nakamura J. Oguchi A. Asada M. Suzuki N.. (2011). Dysfunction of fibroblasts of extrarenal origin underlies renal fibrosis and renal anemia in mice. J. Clin. Invest. 121, 3981–3990. 10.1172/JCI57301 21911936 PMC3195468

[B2] Bechtel W. McGoohan S. Zeisberg E. M. Muller G. A. Kalbacher H. Salant D. J.. (2010). Methylation determines fibroblast activation and fibrogenesis in the kidney. Nat. Med. 16, 544–550. 10.1038/nm.2135 20418885 PMC3106179

[B3] Bernhardt W. M. Wiesener M. S. Scigalla P. Chou J. Schmieder R. E. Gunzler V.. (2010). Inhibition of prolyl hydroxylases increases erythropoietin production in ESRD. J. Am. Soc. Nephrol. 21, 2151–2156. 10.1681/ASN.2010010116 21115615 PMC3014028

[B4] Boor P. Floege J. (2012). The renal (myo-)fibroblast: a heterogeneous group of cells. Nephrol. Dial. Transplant. 27, 3027–3036. 10.1093/ndt/gfs296 22851626

[B5] Boutin A. T. Weidemann A. Fu Z. Mesropian L. Gradin K. Jamora C.. (2008). Epidermal sensing of oxygen is essential for systemic hypoxic response. Cell 133, 223–334. 10.1016/j.cell.2008.02.038 18423195 PMC2849644

[B6] Broekema M. Harmsen M. C. Koerts J. A. van Kooten T. G. Navis G. van Luyn M. J.. (2007). Tubular engraftment and myofibroblast differentiation of recipient-derived cells after experimental kidney transplantation. Transplantation 84, 1003–1011. 10.1097/01.tp.0000285298.05242.f1 17989606

[B7] Bunn H. F. (2013). Erythropoietin. Cold Spring Harb. Perspect. Med. 3:a011619. 10.1101/cshperspect.a011619 23457296 PMC3579209

[B8] Campanholle G. Mittelsteadt K. Nakagawa S. Kobayashi A. Lin S. L. Gharib S. A.. (2013). TLR-2/TLR-4 TREM-1 signaling pathway is dispensable in inflammatory myeloid cells during sterile kidney injury. PLoS ONE 8:e68640. 10.1371/journal.pone.0068640 23844229 PMC3700949

[B9] Chiang C. K. Tanaka T. Inagi R. Fujita T. Nangaku M. (2011). Indoxyl sulfate, a representative uremic toxin, suppresses erythropoietin production in a HIF-dependent manner. Lab Invest. 91, 1564–1571. 10.1038/labinvest.2011.114 21863063

[B10] Dimke H. Sparks M. A. Thomson B. R. Frische S. Coffman T. M. Quaggin S. E. (2015). Tubulovascular cross-talk by vascular endothelial growth factor a maintains peritubular microvasculature in kidney. J. Am. Soc. Nephrol. 26, 1027–1038. 10.1681/ASN.2014010060 25385849 PMC4413754

[B11] Ding M. Cui S. Li C. Jothy S. Haase V. Steer B. M.. (2006). Loss of the tumor suppressor Vhlh leads to upregulation of Cxcr4 and rapidly progressive glomerulonephritis in mice. Nat. Med. 12, 1081–1087. 10.1038/nm1460 16906157

[B12] Eckardt K. U. Koury S. T. Tan C. C. Schuster S. J. Kaissling B. Ratcliffe P. J.. (1993). Distribution of erythropoietin producing cells in rat kidneys during hypoxic hypoxia. Kidney Int. 43, 815–823. 10.1038/ki.1993.115 8479117

[B13] Fahling M. Mathia S. Paliege A. Koesters R. Mrowka R. Peters H.. (2013). Tubular von Hippel-Lindau knockout protects against rhabdomyolysis-induced AKI. J. Am. Soc. Nephrol. 24, 1806–1819. 10.1681/asn.2013030281 23970125 PMC3810090

[B14] Fioretto P. Sutherland D. E. Najafian B. Mauer M. (2006). Remodeling of renal interstitial and tubular lesions in pancreas transplant recipients. Kidney Int. 69, 907–912. 10.1038/sj.ki.5000153 16518350

[B15] Friedman S. L. Sheppard D. Duffield J. S. Violette S. (2013). Therapy for fibrotic diseases: nearing the starting line. Sci. Transl. Med. 5:167sr1. 10.1126/scitranslmed.3004700 23303606

[B16] Fujiu K. Manabe I. Nagai R. (2011). Renal collecting duct epithelial cells regulate inflammation in tubulointerstitial damage in mice. J. Clin. Invest. 121, 3425–3441. 10.1172/JCI57582 21821915 PMC3163964

[B17] Galson D. L. Tsuchiya T. Tendler D. S. Huang L. E. Ren Y. Ogura T.. (1995). The orphan receptor hepatic nuclear factor 4 functions as a transcriptional activator for tissue-specific and hypoxia-specific erythropoietin gene expression and is antagonized by EAR3/COUP-TF1. Mol. Cell. Biol. 15, 2135–2144. 7891708 10.1128/mcb.15.4.2135 PMC230441

[B18] Goldberg M. A. Glass G. A. Cunningham J. M. Bunn H. F. (1987). The regulated expression of erythropoietin by two human hepatoma cell lines. Proc. Natl. Acad. Sci. U.S.A. 84, 7972–7976. 10.1073/pnas.84.22.7972 2825172 PMC299458

[B19] Grgic I. Campanholle G. Bijol V. Wang C. Sabbisetti V. S. Ichimura T.. (2012). Targeted proximal tubule injury triggers interstitial fibrosis and glomerulosclerosis. Kidney Int. 82, 172–183. 10.1038/ki.2012.20 22437410 PMC3480325

[B20] Gruber M. Hu C. J. Johnson R. S. Brown E. J. Keith B. Simon M. C. (2007). Acute postnatal ablation of Hif-2alpha results in anemia. Proc. Natl. Acad. Sci. U.S.A. 104, 2301–2306. 10.1073/pnas.0608382104 17284606 PMC1892942

[B21] Haase V. H. (2010). Hypoxic regulation of erythropoiesis and iron metabolism. Am. J. Physiol. Renal. Physiol. 299, F1–F13. 10.1152/ajprenal.00174.2010 20444740 PMC2904169

[B22] Higgins D. F. Kimura K. Bernhardt W. M. Shrimanker N. Akai Y. Hohenstein B.. (2007). Hypoxia promotes fibrogenesis *in vivo* via HIF-1 stimulation of epithelial-to-mesenchymal transition. J. Clin. Invest. 117, 3810–3820. 10.1172/jci30487 18037992 PMC2082142

[B23] Humphreys B. D. Lin S. L. Kobayashi A. Hudson T. E. Nowlin B. T. Bonventre J. V.. (2010). Fate tracing reveals the pericyte and not epithelial origin of myofibroblasts in kidney fibrosis. Am. J. Pathol. 176, 85–97. 10.2353/ajpath.2010.090517 20008127 PMC2797872

[B24] Humphreys B. D. Xu F. Sabbisetti V. Grgic I. Naini S. M. Wang N.. (2013). Chronic epithelial kidney injury molecule-1 expression causes murine kidney fibrosis. J. Clin. Invest. 123, 4023–4035. 10.1172/JCI45361 23979159 PMC3755983

[B25] Imagawa S. Goldberg M. A. Doweiko J. Bunn H. F. (1991). Regulatory elements of the erythropoietin gene. Blood 77, 278–285. 1985694

[B26] Inomata S. Itoh M. Imai H. Sato T. (1997). Serum levels of erythropoietin as a novel marker reflecting the severity of diabetic nephropathy. Nephron 75, 426–430. 10.1159/000189580 9127329

[B27] Iwano M. Plieth D. Danoff T. M. Xue C. Okada H. Neilson E. G. (2002). Evidence that fibroblasts derive from epithelium during tissue fibrosis. J. Clin. Invest. 110, 341–350. 10.1172/JCI0215518 12163453 PMC151091

[B28] Jacobs K. Shoemaker C. Rudersdorf R. Neill S. D. Kaufman R. J. Mufson A.. (1985). Isolation and characterization of genomic and cDNA clones of human erythropoietin. Nature 313, 806–810. 10.1038/313806a0 3838366

[B29] Jelkmann W. (2002). The enigma of the metabolic fate of circulating erythropoietin (Epo) in view of the pharmacokinetics of the recombinant drugs rhEpo and NESP. Eur. J. Haematol. 69, 265–274. 10.1034/j.1600-0609.2002.02813.x 12460230

[B30] Kang H. M. Ahn S. H. Choi P. Ko Y. A. Han S. H. Chinga F.. (2015). Defective fatty acid oxidation in renal tubular epithelial cells has a key role in kidney fibrosis development. Nat. Med. 21, 37–46. 10.1038/nm.3762 25419705 PMC4444078

[B31] Kapitsinou P. P. Sano H. Michael M. Kobayashi H. Davidoff O. Bian A.. (2014). Endothelial HIF-2 mediates protection and recovery from ischemic kidney injury. J. Clin. Invest. 124, 2396–2409. 10.1172/JCI69073 24789906 PMC4092875

[B32] Kisseleva T. Cong M. Paik Y. Scholten D. Jiang C. Benner C.. (2012). Myofibroblasts revert to an inactive phenotype during regression of liver fibrosis. Proc. Natl. Acad. Sci. U.S.A. 109, 9448–9453. 10.1073/pnas.1201840109 22566629 PMC3386114

[B33] Kobayashi H. Gilbert V. Liu Q. Kapitsinou P. P. Unger T. L. Rha J.. (2012). Myeloid cell-derived hypoxia-inducible factor attenuates inflammation in unilateral ureteral obstruction-induced kidney injury. J. Immunol. 188, 5106–5115. 10.4049/jimmunol.1103377 22490864 PMC3345098

[B34] Koesters R. Kaissling B. Lehir M. Picard N. Theilig F. Gebhardt R.. (2010). Tubular overexpression of transforming growth factor-beta1 induces autophagy and fibrosis but not mesenchymal transition of renal epithelial cells. Am. J. Pathol. 177, 632–643. 10.2353/ajpath.2010.091012 20616344 PMC2913362

[B35] Koury M. J. (2005). Erythropoietin: the story of hypoxia and a finely regulated hematopoietic hormone. Exp. Hematol. 33, 1263–1270. 10.1016/j.exphem.2005.06.031 16263408

[B36] Koury S. T. Koury M. J. Bondurant M. C. Caro J. Graber S. E. (1989). Quantitation of erythropoietin-producing cells in kidneys of mice by *in situ* hybridization: correlation with hematocrit, renal erythropoietin mRNA, and serum erythropoietin concentration. Blood 74, 645–651. 2752138

[B37] Kramann R. Schneider R. K. Dirocco D. P. Machado F. Fleig S. Bondzie P. A.. (2015). Perivascular gli1(+) progenitors are key contributors to injury-induced organ fibrosis. Cell Stem Cell 16, 51–66. 10.1016/j.stem.2014.11.004 25465115 PMC4289444

[B38] Kuo C. C. Lee C. T. Chuang C. H. Su Y. Chen J. B. (2005). Recombinant human erythropoietin independence in chronic hemodialysis patients: clinical features, iron homeostasis and erythropoiesis. Clin. Nephrol. 63, 92–97. 10.5414/CNP63092 15730050

[B39] LeBleu V. S. Taduri G. O'Connell J. Teng Y. Cooke V. G. Woda C.. (2013). Origin and function of myofibroblasts in kidney fibrosis. Nat. Med. 19, 1047–1053. 10.1038/nm.3218 23817022 PMC4067127

[B40] Li L. Zepeda-Orozco D. Black R. Lin F. (2010). Autophagy is a component of epithelial cell fate in obstructive uropathy. Am. J. Pathol. 176, 1767–1778. 10.2353/ajpath.2010.090345 20150430 PMC2843468

[B41] Lin F. K. Suggs S. Lin C. H. Browne J. K. Smalling R. Egrie J. C.. (1985). Cloning and expression of the human erythropoietin gene. Proc. Natl. Acad. Sci. U.S.A. 82, 7580–7584. 10.1073/pnas.82.22.7580 3865178 PMC391376

[B42] Lin S. L. Castaño A. P. Nowlin B. T. Lupher M. L. Jr. Duffield J. S. (2009). Bone marrow Ly6Chigh monocytes are selectively recruited to injured kidney and differentiate into functionally distinct populations. J. Immunol. 183, 6733–6743. 10.4049/jimmunol.0901473 19864592

[B43] Lin S. L. Kisseleva T. Brenner D. A. Duffield J. S. (2008). Pericytes and perivascular fibroblasts are the primary source of collagen-producing cells in obstructive fibrosis of the kidney. Am. J. Pathol. 173, 1617–1627. 10.2353/ajpath.2008.080433 19008372 PMC2626374

[B44] Mack M. Yanagita M. (2014). Origin of myofibroblasts and cellular events triggering fibrosis. Kidney Int. 10.1038/ki.2014.287 25162398

[B45] Madan A. Lin C. Hatch S. L. Curtin P. T. (1995). Regulated basal, inducible, and tissue-specific human erythropoietin gene-expression in transgenic mice requires multiple cis DNA-sequences. Blood 85, 2735–2741. 7742534

[B46] Mahon P. C. Hirota K. Semenza G. L. (2001). FIH-1: a novel protein that interacts with HIF-1alpha and VHL to mediate repression of HIF-1 transcriptional activity. Genes Dev. 15, 2675–2686. 10.1101/gad.924501 11641274 PMC312814

[B47] Makita T. Hernandez-Hoyos G. Chen T. H. Wu H. Rothenberg E. V. Sucov H. M. (2001). A developmental transition in definitive erythropoiesis: erythropoietin expression is sequentially regulated by retinoic acid receptors and HNF4. Genes Dev. 15, 889–901. 10.1101/gad.871601 11297512 PMC312661

[B48] Masson N. Singleton R. S. Sekirnik R. Trudgian D. C. Ambrose L. J. Miranda M. X.. (2012). The FIH hydroxylase is a cellular peroxide sensor that modulates HIF transcriptional activity. EMBO Rep. 13, 251–257. 10.1038/embor.2012.9 22310300 PMC3323130

[B49] Maxwell P. H. Ferguson D. J. Nicholls L. G. Johnson M. H. Ratcliffe P. J. (1997). The interstitial response to renal injury: fibroblast-like cells show phenotypic changes and have reduced potential for erythropoietin gene expression. Kidney Int. 52, 715–724. 10.1038/ki.1997.387 9291192

[B49a] Maxwell P. H. Osmond M. K. Pugh C. W. Heryet A. Nicholls L. G. Tan C. C.. (1993). Identification of the renal erythropoietin-producing cells using transgenic mice. Kidney Int. 44, 1149–1162. 8264149 10.1038/ki.1993.362

[B50] Miyake T. Kung C. K. Goldwasser E. (1977). Purification of human erythropoietin. J. Biol. Chem. 252, 5558–5564. 18467

[B51] Miyata T. Suzuki N. van Ypersele de Strihou C. (2013). Diabetic nephropathy: are there new and potentially promising therapies targeting oxygen biology? Kidney Int. 84, 693–702. 10.1038/ki.2013.74 23486514

[B52] Nakano Y. Imagawa S. Matsumoto K. Stockmann C. Obara N. Suzuki N.. (2004). Oral administration of K-11706 inhibits GATA binding activity, enhances hypoxia-inducible factor 1 binding activity, and restores indicators in an *in vivo* mouse model of anemia of chronic disease. Blood 104, 4300–4307. 10.1182/blood-2004-04-1631 15328158

[B53] Nangaku M. (2006). Chronic hypoxia and tubulointerstitial injury: a final common pathway to end-stage renal failure. J. Am. Soc. Nephrol. 17, 17–25. 10.1681/ASN.2005070757 16291837

[B54] Noguchi C. T. Wang L. Rogers H. M. Teng R. Jia Y. (2008). Survival and proliferative roles of erythropoietin beyond the erythroid lineage. Expert Rev. Mol. Med. 10:e36. 10.1017/S1462399408000860 19040789 PMC3065109

[B55] Obara N. Suzuki N. Kim K. Nagasawa T. Imagawa S. Yamamoto M. (2008). Repression via the GATA box is essential for tissue-specific erythropoietin gene expression. Blood 111, 5223–5232. 10.1182/blood-2007-10-115857 18202227

[B56] Pagel H. Jelkmann W. Weiss C. (1990). Erythropoietin production in the isolated perfused kidney. Biomed. Biochim. Acta. 49, S271–274. 2386516

[B57] Pan X. Suzuki N. Hirano I. Yamazaki S. Minegishi N. Yamamoto M. (2011). Isolation and characterization of renal erythropoietin-producing cells from genetically produced anemia mice. PLoS ONE 6:e25839. 10.1371/journal.pone.0025839 22022454 PMC3191152

[B58] Paragas N. Qiu A. Zhang Q. Samstein B. Deng S. X. Schmidt-Ott K. M.. (2011). The Ngal reporter mouse detects the response of the kidney to injury in real time. Nat. Med. 17, 216–222. 10.1038/nm.2290 21240264 PMC3059503

[B59] Quaggin S. E. Kapus A. (2011). Scar wars: mapping the fate of epithelial–mesenchymal–myofibroblast transition. Kidney Int. 80, 41–50. 10.1038/ki.2011.77 21430641

[B60] Rankin E. B. Tomaszewski J. E. Haase V. H. (2006). Renal cyst development in mice with conditional inactivation of the von Hippel-Lindau tumor suppressor. Cancer Res. 66, 2576–2583. 10.1158/0008-5472.CAN-05-3241 16510575 PMC3514875

[B61] Ratcliffe P. J. (2013). Oxygen sensing and hypoxia signalling pathways in animals: the implications of physiology for cancer. J. Physiol. 591, 2027–2042. 10.1113/jphysiol.2013.251470 23401619 PMC3634517

[B62] Remy I. Wilson I. A. Michnick S. W. (1999). Erythropoietin receptor activation by a ligand-induced conformation change. Science 283, 990–993. 10.1126/science.283.5404.990 9974393

[B63] Roufosse C. Bou-Gharios G. Prodromidi E. Alexakis C. Jeffery R. Khan S.. (2006). Bone marrow-derived cells do not contribute significantly to collagen I synthesis in a murine model of renal fibrosis. J. Am. Soc. Nephrol. 17, 775–782. 10.1681/ASN.2005080795 16467445

[B64] Schrimpf C. Xin C. Campanholle G. Gill S. E. Stallcup W. Lin S. L.. (2012). Pericyte TIMP3 and ADAMTS1 modulate vascular stability after kidney injury. J. Am. Soc. Nephrol. 23, 868–883. 10.1681/ASN.2011080851 22383695 PMC3338296

[B65] Schwartz D. I. Pierratos A. Richardson R. M. Fenton S. S. Chan C. T. (2005). Impact of nocturnal home hemodialysis on anemia management in patients with end-stage renal disease. Clin. Nephrol. 63, 202–208. 10.5414/CNP63202 15786821

[B66] Semenza G. L. (2011). Oxygen sensing, homeostasis, and disease. N. Engl. J. Med. 365, 537–547. 10.1056/NEJMra1011165 21830968

[B67] Semenza G. L. Koury S. T. Nejfelt M. K. Gearhart J. D. Antonarakis S. E. (1991a). Cell-type-specific and hypoxia-inducible expression of the human erythropoietin gene in transgenic mice. Proc. Natl. Acad. Sci. U.S.A. 88, 8725–8729. 10.1073/pnas.88.19.8725 1924331 PMC52582

[B68] Semenza G. L. Nejfelt M. K. Chi S. M. Antonarakis S. E. (1991b). Hypoxia-inducible nuclear factors bind to an enhancer element located 3′ to the human erythropoietin gene. Proc. Natl. Acad. Sci. U.S.A. 88, 5680–5684. 10.1073/pnas.88.13.5680 2062846 PMC51941

[B69] Souma T. Nezu M. Nakano D. Yamazaki S. Hirano I. Sekine H. (in press). Erythropoietin synthesis in renal myofibroblasts is restored by activation of hypoxia signaling. J. Am. Soc. Nephrol. 10.1681/ASN.2014121184 PMC4731118 26054543

[B70] Souma T. Yamazaki S. Moriguchi T. Suzuki N. Hirano I. Pan X.. (2013). Plasticity of renal erythropoietin-producing cells governs fibrosis. J. Am. Soc. Nephrol. 24, 1599–1616. 10.1681/ASN.2013010030 23833259 PMC3785278

[B71] Suzuki N. (2015). Erythropoietin gene expression: developmental-stage specificity, cell-type specificity, and hypoxia inducibility. Tohoku J. Exp. Med. 235, 233–240. 10.1620/tjem.235.233 25786542

[B72] Suzuki N. Hirano I. Pan X. Minegishi N. Yamamoto M. (2013). Erythropoietin production in neuroepithelial and neural crest cells during primitive erythropoiesis. Nat. Commun. 4:2902. 10.1038/ncomms3902 24309470

[B73] Suzuki N. Obara N. Pan X. Watanabe M. Jishage K. Minegishi N.. (2011). Specific contribution of the erythropoietin gene 3′ enhancer to hepatic erythropoiesis after late embryonic stages. Mol. Cell. Biol. 31, 3896–3905. 10.1128/MCB.05463-11 21746884 PMC3165733

[B74] Suzuki N. Obara N. Yamamoto M. (2007). Use of gene-manipulated mice in the study of erythropoietin gene expression. Methods Enzymol. 435, 157–177. 10.1016/S0076-6879(07)35009-X 17998054

[B75] Suzuki N. Suwabe N. Ohneda O. Obara N. Imagawa S. Pan X.. (2003). Identification and characterization of 2 types of erythroid progenitors that express GATA-1 at distinct levels. Blood 102, 3575–3583. 10.1182/blood-2003-04-1154 12893747

[B76] Takeda A. Toda T. Shinohara S. Mogi Y. Matsui N. (2002). Factors contributing to higher hematocrit levels in hemodialysis patients not receiving recombinant human erythropoietin 1. Am. J. Kidney Dis. 40, 104–109. 10.1053/ajkd.2002.33918 12087567

[B77] Takeda K. Aguila H. L. Parikh N. S. Li X. Lamothe K. Duan L. J.. (2008). Regulation of adult erythropoiesis by prolyl hydroxylase domain proteins. Blood 111, 3229–3235. 10.1182/blood-2007-09-114561 18056838 PMC2265459

[B78] Tanimoto K. Makino Y. Pereira T. Poellinger L. (2000). Mechanism of regulation of the hypoxia-inducible factor-1 alpha by the von Hippel-Lindau tumor suppressor protein. EMBO J. 19, 4298–4309. 10.1093/emboj/19.16.4298 10944113 PMC302039

[B79] Tarumoto T. Imagawa S. Ohmine K. Nagai T. Higuchi M. Imai N.. (2000). N(G)-monomethyl-L-arginine inhibits erythropoietin gene expression by stimulating GATA-2. Blood 96, 1716–1722. 10961869

[B80] Tian Y. M. Yeoh K. K. Lee M. K. Eriksson T. Kessler B. M. Kramer H. B.. (2011). Differential sensitivity of hypoxia inducible factor hydroxylation sites to hypoxia and hydroxylase inhibitors. J. Biol. Chem. 286, 13041–13051. 10.1074/jbc.M110.211110 21335549 PMC3075650

[B81] Wu H. Liu X. Jaenisch R. Lodish H. F. (1995). Generation of committed erythroid BFU-E and CFU-E progenitors does not require erythropoietin or the erythropoietin receptor. Cell 83, 59–67. 10.1016/0092-8674(95)90234-1 7553874

[B82] Yamazaki S. Souma T. Hirano I. Pan X. Minegishi N. Suzuki N.. (2013). A mouse model of adult-onset anaemia due to erythropoietin deficiency. Nat. Commun. 4:1950. 10.1038/ncomms2950 23727690

[B83] Yin H. Blanchard K. L. (2000). DNA methylation represses the expression of the human erythropoietin gene by two different mechanisms. Blood 95, 111–119. 10607693

[B84] Zeisberg E. M. Potenta S. E. Sugimoto H. Zeisberg M. Kalluri R. (2008). Fibroblasts in kidney fibrosis emerge via endothelial-to-mesenchymal transition. J. Am. Soc. Nephrol. 19, 2282–2287. 10.1681/ASN.2008050513 18987304 PMC2588112

[B85] Zeisberg M. Hanai J. Sugimoto H. Mammoto T. Charytan D. Strutz F.. (2003). BMP-7 counteracts TGF-beta1-induced epithelial-to-mesenchymal transition and reverses chronic renal injury. Nat. Med. 9, 964–968. 10.1038/nm888 12808448

[B86] Zhang N. Fu Z. Linke S. Chicher J. Gorman J. J. Visk D.. (2010). The asparaginyl hydroxylase factor inhibiting HIF-1alpha is an essential regulator of metabolism. Cell Metab. 11, 364–378. 10.1016/j.cmet.2010.03.001 20399150 PMC2893150

